# Association between Homophobia and Sociodemographic Characteristics in Health Workers in Southern Chile

**DOI:** 10.3390/ijerph192113749

**Published:** 2022-10-22

**Authors:** Oscar Oyarce-Vildósola, Alejandra Rodríguez-Fernández, Eduard Maury-Sintjago

**Affiliations:** 1 Master’s Degree Program in Public Health, Faculty of Health and Food Sciences, Universidad del Bío-Bío, Chillan 3780000, Chile; 2 Department of Nutrition and Public Health, Universidad del Bío-Bío, Chillan 3780000, Chile; 3 Auxology, Bioanthropology, and Ontogeny Research Group (GABO), Universidad del Bío-Bío, Chillan 3780000, Chile

**Keywords:** homophobia, health workers, LGBTIQ+

## Abstract

Homophobic attitude in health workers is a social determinant in the health of the homosexual population because it affects healthcare and its access and equity. The objective was to determine the relationship between the level of homophobia and sociodemographic characteristics of primary health workers in southern Chile. This was an analytical cross-sectional study. The sample consisted of 491 public servants from health centers in southern Chile. The attitudes toward lesbians and gay men (ATLG) scale was applied, and a binary logistic regression model was performed to evaluate the association. The study participants were predominantly women (76.0%), under 40 years of age (63.5%), heterosexuals (93.5%), and unpartnered (68.2%) but with children (61.7%), and they also had an educational attainment ≥ 12 years (66.6%). About 87.6% of the participants held healthcare positions, and the majority were religious believers (74.3%) and had a centrist political affiliation (51.0%). Results indicated that 34% of the participants exhibited homophobic attitudes; there were statistically significant differences with respect to age, number of children, educational attainment, religion, and political affiliation (*p* < 0.01). These were higher in individuals ≥40 years of age, with ≥3 children, with educational attainment ≥ 12 years, holding a healthcare position, who were religious believers, and who had right-wing political affiliation.

## 1. Introduction

Homophobia has been defined as the negative attitude, aversion, rejection, intolerance, and fear toward homosexuals, which is primarily based on a system of beliefs, values, or ideological principles of the hegemonic heteronormativity model [1]. Its main consequence is the discrimination suffered by homosexuals in the family, educational, work, and social settings [2].

Homophobia is a scourge on the health of homosexuals, affecting both physical and mental health. In this regard, studies indicate that there is a higher prevalence of cardiovascular and endocrine diseases in people who suffer from homophobia [3,4], mediated, in large part, by the stress of minorities, which has an additive effect on cardiometabolic reactivity beyond the consequences of ordinary social stressors [3,4,5,6]. Furthermore, the literature indicates that people who experience victimization based on homophobic attitudes are more at risk for mental health problems, such as depression, anxiety, and suicidal tendencies [7,8].

On the other hand, prejudice toward the homosexual community is rooted in institutionalized, state-sponsored, social, and internalized homophobic attitudes, which are based on culture, religion, and the political-economic system, among others [9]. Structural stigma toward homosexuals is rooted in the development of laws, health policies, access, and delivery of resources. Pachankis et al. [10], in their study in 38 European countries, showed that, for example, the risk of becoming ill with HIV is explained more by national homophobic attitudes than by personal failure.

Zeeman et al. [11] conducted a systematic review of health and healthcare inequalities. They indicated that lesbian, gay, bisexual, trans, intersex, queer, and other non-normative sexual identities (LGBTIQ+) are more likely to experience healthcare inequalities due to prevailing heteronormativity, discrimination, and stigmatization of the community. Moreover, there are differences within the same discriminated groups according to gender, age, income, and disability. 

Homophobic attitudes in Latin America have been changing in recent decades [12]. Chile is no exception, and a greater flexibility in attitudes toward sexual minorities has been noted; it can, therefore, be stated that the country is less homophobic now than a few years ago [13]. Barrientos [14] analyzed homophobia in Chile and found that the country ranked third, behind Argentina and Uruguay, for its more positive attitudes toward homosexuals. However, the percentage of tolerance toward homosexuality at the population level is still low.

Therefore, Chilean health services have focused their efforts on implementing units that advise primary, secondary, and tertiary health centers on how to provide discrimination-free care to the LGBTIQ+ community [15,16]. Thus, mechanisms have been implemented that seek to provide comprehensive care designed for transsexual beneficiaries who wish to begin their gender transition in a safe manner, with counseling by specifically trained professionals and with an approach that does not consider this population as individuals with mental health pathologies that require reconversion interventions [15]. 

The literature also supports the need for quality and nondiscriminatory care to promote the self-care of users who, for various reasons, prefer not to go to health centers [17]. To achieve this, it is of utmost importance to know the local reality regarding this problem that affects public servants and users of the primary healthcare system. This is carried out by detecting the level of homophobia among public servants using validated instruments and the analysis of associated conditions, which raise awareness of the risk of exhibiting attitudes that could be against national regulations concerning the care of the homosexual population [18]. 

Homophobia can be conditioned by different sociodemographic factors such as age, civil status, or educational attainment [19,20]. The negative attitude toward homosexuality is reinforced by religious ideologies [21]. Some studies have demonstrated a decrease among professionals in different fields regarding negative attitudes toward homosexuals. The study by Rodríguez-Otero [1] included Spanish psychologists, social workers, and educational psychologists, which showed the prevalence of mostly positive attitudes toward homosexuals. Penna and Mateos [22] and Maury-Sintjago et al. [23] reported similar results for education and nursing students, respectively.

It is necessary to investigate the current situation in the family health centers (CESFAM) in southern Chile to identify existing knowledge gaps, inequalities, and access barriers related to the care of homosexual users of the primary healthcare system. Therefore, the objective was to determine the relationship between the level of homophobia and sociodemographic characteristics of primary health workers in southern Chile.

## 2. Materials and Methods

### 2.1. Study Design

This was an analytical cross-sectional study. 

### 2.2. Samples

The probabilistic sample consisted of 491 public servants belonging to six family health centers (CESFAM) in southern Chile, which was made up mostly of women (76%) due to the occupational characteristics of the health sector. The sample size was calculated based on a stratified sampling by family health center and position with 3% precision and 95% confidence interval. The following inclusion criteria were applied: public servants with current contracts, of legal age, both sexes, and who had given informed consent. Participants were excluded if they were on any type of leave (medical leave, administrative leave, or others) or did not complete the information contained in the data collection instruments. 

This study was reviewed and approved by the Ethics and Biosecurity Committee of the Universidad del Bío-Bío (Concepción, Chile). All participants gave informed consent to be part of the study. The procedures used in the investigation were according to the ethical principles of the Declaration of Helsinki and the Council for International Organizations of Medical Sciences (CIOMS) [24]. 

### 2.3. Study Variables 

A questionnaire was applied to collect the following data: age, sex, number of children, sexual orientation, civil status, religion, political affiliation, educational attainment, and position. 

The attitudes toward lesbians and gay men (ATLG) scale developed by Herek [25] was applied to evaluate public servant attitudes. This scale is based on reported affective responses of heterosexual men and women toward homosexual individuals. The instrument consists of 20 items with a five-point Likert scale (from 1 = strongly disagree to 5 = strongly agree). Of the 20 items, 10 evaluate the attitude toward gay men and 10 toward lesbians. A homophobic attitude was defined as a score ≥ 60 points [26]. The internal consistency of the scale is high (α > 0.80) and has an attitudinal condemnation–tolerance dimension. The ATLG scale has been validated in the Chilean population by Cárdenas and Barrientos [18]; it was confirmed in our study with a score of 0.9 according to Cronbach’s alpha test. 

### 2.4. Statistical Analysis

The normal distribution of the numerical variables was verified by the Shapiro–Wilk test. Data were expressed as means, standard deviation, percentages, and absolute frequencies according to their nature and normality. The *t*-test was used to compare the quantitative variables in two groups, whereas one-way ANOVA was used for more than two groups. The variables were analyzed by the chi-squared test. Statistical significance was established as α < 0.05. A binary logistic regression model was performed to evaluate the association of the predictive variables with the response. All statistical analyses were performed with STATA version 16.0 software (StataCorp LP, College Station, TX, USA) at α = 0.05 level of significance.

## 3. Results

Study participants were predominantly women (76.0%), under 40 years of age (63.5%), heterosexual (93.5%), and mostly unpartnered (68.2%) but with children (61.7%). Educational attainment was ≥12 years (73.5%). About 87.6% of the participants held healthcare positions. Finally, most subjects were religious believers (74.3%) and had a centrist political affiliation (51.0%) (Table 1).

There were some variations in the mean scores obtained for each item of the ATLG scale. The items with the highest scores were “If I knew my child was homosexual, I would not be depressed (4.6 ± 3.1)” and “Male homosexuality is a natural expression of male sexuality (4.2 ± 2.8)”. Meanwhile, the items with the lowest scores were “Lesbians are sick (1.5 ± 1.3)” and “I think that male homosexuals are revolting (1.6 ± 1.4)”. The average total score of the scale was 50.7 ± 25.2. When analyzing the overall scores for homophobia categorization, 34% of the participants exhibited homophobic attitudes. 

Table 2 shows the relationship between the sociodemographic variables and homophobic attitude of the study subjects. Significant statistical differences were found for age, number of children, educational attainment, position, religion, and political affiliation (*p* < 0.01). Sex, sexual orientation, and domestic partnership were unrelated (*p* > 0.05).

The results of the logistic regression model indicated that homophobia increased almost twofold in individuals with children compared with those with no children (OR = 17; CI: 1.03–2.66). The same situation occurred for participants with low educational attainment (OR = 2.1; CI: 1.27–3.52), who were religious believers (OR = 2.2; CI: 1.25–3.61), and who had right-wing political affiliation (OR = 2.3; CI = 1.24–4.23). This was also the case for participants holding an administrative position in which the risk of homophobia could be as much as five times higher at the upper end of the interval when compared with health workers (OR = 2.5; CI: 1.35–4.7) (Table 3). 

## 4. Discussion 

The study of the level of homophobia in healthcare institutions is an indispensable tool for people management because it enables the identification of discriminatory attitudes or behaviors toward individuals belonging to the LGBTIQ+ community. It also identifies the impact that these attitudes and behaviors can have on the quality of care, access to quality health services, and health equity. 

Clear differences associated with the sex of respondents have been identified in several studies and meta-analyses, which showed that being a woman was a factor that decreased the frequency of homophobic attitudes; women also felt more comfortable working with homosexual men as opposed to the male population, which repeatedly showed higher rates of negative attitudes toward the LGBTIQ+ population [27,28,29,30]. Studies of Latin American adolescents had similar results; there were higher rates of homophobic attitudes in male Chilean and female Colombian adolescents compared with male Colombian and female Chilean adolescents [31]. 

Most of our study participants were women (76%), which is similar to the numbers reported by the health system. Despite the difference in proportionality by sex, our data showed that there was no significant difference in the sex variable among the respondents. When analyzing age and attitudes toward homosexuality, a significant difference was identified in respondents over 40 years of age, who indicated a greater likelihood of having more negative attitudes toward the LGBTIQ+ community. This concurs with studies that have identified that being older is generally associated with more negative attitudes, and that acceptance of homosexuality increases as the age of respondents decreases [22,32]. However, it is necessary to analyze whether these associations are due to generational differences toward homosexuality rather than to the chronological age of the respondents [33]. 

The civil status variable provided values with no significant differences with respect to homophobic attitudes. This contrasts with various studies that identified that being married or widowed was among a group of conditions related to more homophobic and discriminatory attitudes [34,35]. Homophobic attitudes based on civil status largely depend on sociocultural aspects and social prejudice. Therefore, in a study of Turkish professionals, Yertutanol et al. [36] reported that single subjects had more homophobic attitudes than other civil statuses. Despite the fact that homosexuality is legal in Turkey since 1858, religious factors continue to generate major challenges for the homosexual community [37].

Significant differences were identified when analyzing the number of children of the respondents; public servants with more children had a high possibility of exhibiting homophobic attitudes. This concurs with several studies that have linked large families to this tendency [35,36,37]. 

Our findings revealed that participants with lower educational attainment were more likely to have homophobic attitudes; this is consistent with previous studies [35,36,37]. In their study with the national representation of the General Social Survey (GSS) in the United States between 1988 and 1994, Ohlander et al. [38] showed that each additional educational attainment level decreased the probability of disapproving homosexuality by 40% (exp(β) = 0.6, *p* < 0.01). This is similar to our results, which revealed that lower educational attainment increased homophobic attitudes by as much as 3.5 times (OR = 2.1; CI: 1.27–3.52). Education is usually associated with attitudes that are more tolerant toward homosexual individuals, particularly among respondents with higher education; this can promote reasoning that fosters greater tolerance and thus less homophobic attitudes and homonegativity [28,38]. Likewise, when analyzing the position of the public servants within the facility, there were significant differences based on their functions, and those belonging to healthcare positions showed less homophobic attitudes.

Studies that have examined the attitudes of healthcare workers toward individuals belonging to the LGBTIQ+ community have shown varied results. A large number of healthcare workers were unfamiliar with this community, and nearly 30% did not want to provide care to these individuals or were reluctant to do so; they exhibited above-average homophobic attitudes, which prevail in society and in healthcare [37,39,40].

Religion has been extensively studied by various authors, and the presence of homophobic attitudes has generally been associated with a higher degree of religiosity [34,41,42]. We found that being a religious believer increased the probability of homophobic attitudes by as much as 3.6 times in the upper limit (OR = 2.2; CI: 1.25–3.61). This is consistent with the study by Adamczyk and Pitt [35] with 60,047 adults from 40 countries. They showed that not having a religion decreased the negative attitude toward homosexuality (β = −0.531; *p* < 0.01), whereas having a religious affiliation increased homophobia (β = −0.289; *p* < 0.01) and explained an additional 37% of the variation in attitudes toward homosexuality. This has been previously analyzed and associated with the most conservative societies, religious fundamentalism, and frequent attendance at religious services; non-Catholic Christians (Evangelicals, Jehovah’s Witnesses, Mormons, Adventists, Baptists, and Jews) were highlighted because the most conservative religious teachings were associated with the development of a conservative ethic [27,28,34,37,41,42]. 

When evaluating the impact of political affiliation, individuals who identify themselves as “right-wing” showed stronger homophobic attitudes than the other two political options. This has been reported in several studies that provided evidence that a conservative political tendency was associated with more negative attitudes toward homosexuals [28,43]. Andreescu [44] studied 2139 data of Romanians from the European Social Survey (ESS) and concluded that there was a higher probability of rejecting homosexuality when an individual was conservative (OR = 1.056; CI: 1.009–1.105). This concurs with our results that participants with a right-wing tendency had attitudes that were up to four times more homophobic (OR = 2.3; CI: 1.24–4.23). As with religion, political ideology has been related to constant moral decision-making by which conservatives have emphasized binding principles and liberals have highlighted individualizing principles [45].

Given the significant differences in the data obtained when applying the ATLG survey among healthcare workers, it is therefore necessary to evaluate the problem at the local level because of the impact on the quality of care and the barriers to care that could lead to inappropriate experiences with patients belonging to the LGBTIQ+ community [46].

This research is the first study on homophobia among health workers in Chile, and its importance lies in providing a first approximation of the problem and its associated factors, which could serve as input for a baseline in the formulation of actions by health authorities.

Our study has several limitations that should be considered to correctly interpret the results. It would have been convenient to include qualitative methodologies to identify the real causes underlying homophobic attitudes. Furthermore, the impact that such attitudes have on patient care could have been explored. 

## 5. Conclusions

There is a high prevalence of homophobic attitudes among health workers in southern Chile. Among the factors that most influence this group to reject homosexuality are holding an administrative position, having a conservative political tendency (right-wing), religious affiliation, level of educational attainment, and having children.

## Figures and Tables

**Table 1:** Sociodemographic characterization of study participants.

**Variable**	**n = 491**	**%**
Sex		
Women	373	76.0
Men	118	24.0
Age		
<40 years	312	63.5
≥40 years	179	36.5
Sexual orientation		
Heterosexual	459	93.5
Nonheterosexual	32	6.52
Civil status		
Partnered	156	31.8
Unpartnered	335	68.2
Number of children		
No children	188	38.3
1–2 children	259	52.8
≥3 children	44	8.9
Educational attainment		
<12 years	130	26.5
≥12 years	361	73.5
Position		
Administrative	61	12.4
Healthcare	430	87.6
Religion		
Believer	365	74.3
Nonbeliever	126	25.7
Political affiliation		
Left-wing	113	23.0
Centrist	250	51.0
Right-wing	128	26.0

**Table 2:** Bivariate analysis of presence of homophobic attitudes according to sociodemographic characteristics.

Variable	Homophobic Attitude		*p*
	No	Yes	
Sex			
Women	251 (67.3)	122 (32.7)	0.454
Men	75 (63.7)	43 (36.4)	
Age			
<40 years	225 (72.1)	87 (27.9)	<0.001
≥40 years	101 (56.4)	78 (43.6)	
Sexual orientation			
Heterosexual	301 (65.6)	158 (34.4)	0.146
Nonheterosexual	28 (78.1)	7 (21.9)	
Civil status			
Partnered	95 (60.9)	61 (39.1)	0.078
Unpartnered	231 (69.0)	104 (31.0)	
Number of children			
No children	145 (77.1)	43 (22.9)	<0.001
1–2 children	161 (62.1)	98 (37.8)	
≥3 children	20 (45.5)	24 (54.6)	
Educational attainment			
<12 years	103 (31.6)	223 (68.4)	<0.001
≥12 years	27 (16.4)	138 (83.6)	
Position			
Administrative	303 (70.5)	127 (29.5)	<0.001
Healthcare	23 (37.7)	38 (62.3)	
Religion			
Believer	103 (81.1)	24 (18.9)	<0.001
Nonbeliever	224 (61.37)	141 (33.5)	
Political affiliation			
Left-wing	88 (77.2)	26 (22.8)	0.011
Centrist	163 (65.2)	87 (34.8)	
Right-wing	76 (59.4)	52 (40.6)	

Data expressed as frequency (percentages). Chi-squared test α = 0.05.

**Table 3:** Multiple logistic regression model for homophobia.

Variables	OR_crude_ (95% CI)	OR_ajusted_ (95% CI) *
Having children	2.3 (1.51–3.42)	1.7 (1.03–2.66)
Educational attainment (<12 years)	2.4 (1.47–3.79)	2.1 (1.27–3.52)
Religious believer	2.7 (1.63–4.38)	2.2 (1.25–3.61)
Political affiliation		
Centrist	1.8 (1.07–2.97)	1.5 (0.87–2.63)
Right-wing	2.3 (1.31–4.02)	2.3 (1.24–4.23)
Administrative position	3.9 (2.26–6.88)	2.5 (1.35–4.70)

* Adjusted for sex and age. OR: odds ratio; CI: confidence interval.

## Data Availability

Not applicable.
